# Executive Function Deficits and Borderline Personality Disorder Symptomatology in a Nonclinical Adult Sample: A Latent Variable Analysis

**DOI:** 10.3390/brainsci13020206

**Published:** 2023-01-26

**Authors:** Keisha D. Veerapandian, Gabriel X. D. Tan, Nadyanna M. Majeed, Andree Hartanto

**Affiliations:** 1 School of Social Sciences, Singapore Management University, Singapore 179873, Singapore; 2 Department of Psychology, Faculty of Arts & Social Sciences, National University of Singapore, Singapore 119077, Singapore

**Keywords:** borderline personality disorder, executive functions, BPD symptomatology, inhibitory control, cognitive flexibility, updating-working memory

## Abstract

While borderline personality disorder (BPD) symptomatology has been studied extensively in clinical populations, the mechanisms underlying its manifestation in nonclinical populations remain largely understudied. One aspect of BPD symptomatology in nonclinical populations that has not been well studied is cognitive mechanisms, especially in relation to executive functions. To explore the cognitive mechanisms underlying BPD symptomatology in nonclinical populations, we analysed a large-scale dataset of 233 young adults that were administered with nine executive function tasks and BPD symptomatology assessments. Our structural equation modelling did not find any significant relations between latent factors of executive functions and the severity of BPD symptomatology. Contrary to our hypothesis, our result suggests that deficits in executive functions were not a risk factor for BPD symptomatology in the nonclinical young adult sample.

## 1. Introduction

Borderline personality disorder (BPD) is a serious psychological disorder that presents as one of the most challenging and complicated concerns in the field of psychiatry [1,2,3]. Characterised mainly by emotional dysregulation, impulsivity, and affective instability, BPD severely impairs a person’s quality of life as it results in difficulties in controlling anger, and unstable interpersonal relationships [2,3,4,5]. According to the American Psychiatric Association [6], BPD often manifests through emotional sensitivity, sudden shifts in goals and self-image, and self-damaging behaviours. Furthermore, BPD has been found to be associated with suicide, self-harm, subjective sleep disturbances, neuropsychological dysfunction, additional psychiatric problems, and prevalent stigma in society [6,7,8,9,10,11,12,13]. As one of the most common personality disorders, BPD is estimated to be present in around 1.6% of the general population, 20% of the psychiatric inpatient population, and 10% of the psychiatric outpatient population [14,15]. 

BPD symptomatology has also been suggested to be present in nonclinical populations, and varies across the psychopathological severity continuum [16,17,18]. Although less severe than in clinical populations, symptomatology in nonclinical populations is also beset by a range of psychological dysfunctions including but not limited to maladaptive coping styles, interpersonal stress, romantic dysfunction, and poor psychosocial functioning [16,19,20]. While BPD symptomatology has been studied extensively in clinical populations, the mechanisms underlying its manifestation in nonclinical populations remains largely understudied [9,21,22,23,24]. 

One aspect of BPD symptomatology in nonclinical populations that has not been well studied is its cognitive mechanisms, especially in association with executive functioning. Executive functioning is a multifaceted construct consisting of higher-order cognitive processes that play important roles in regulating thoughts and actions to achieve a goal [25,26]. There are three well-established domains of executive functions: (1) inhibitory control, which is tied to one’s ability to inhibit and control their attention, thoughts, behaviours, and emotions; (2) cognitive flexibility, which is tied to one’s ability to switch between mental sets; and (3) updating-working memory, which is tied to one’s ability to retain information that is no longer in sight and work with it. It is from these three foundational domains that further behavioural strategies—such as planning, adaptation, and volition—can emerge [26]. Consistently, executive functions have been shown to predict many important life outcomes, such as physical health [27,28,29,30], school achievement [31,32,33], emotional well-being [34,35,36,37], social relationships [38,39], and job success [40,41]. Importantly, deficits in executive functions have also been associated with traits associated with prominent BPD symptomatology, such as behavioural impulsivity, emotional reactivity, and emotional dysregulation [22,23,24,33,34,38,42,43,44,45,46,47,48]. This prompts the need to investigate whether deficits in executive functions can potentially be a predictor of BPD symptomatology that is present in nonclinical populations.

The current study thus aims to explore the cognitive mechanisms underlying BPD symptomatology in nonclinical populations. To provide a comprehensive examination of the link between executive functions and BPD symptomatology, we employed a multidimensional approach to executive functions, by measuring the three core domains of executive functions: inhibitory control, cognitive flexibility, and updating-working memory. We also used a latent variable approach to model the construct of executive functioning to overcome issues related to task impurity which may obscure or inflate the true relation between executive functions and BPD symptomatology [25,49,50]. The latent variable approach overcomes this by extracting common variance among the executive function tasks, thereby removing non-executive function processes, giving us a more precise and reliable measure of executive functions. Taken together, we hypothesise that the latent factors of executive functions would predict the severity of BPD symptomatology in nonclinical young adults. 

## 2. Materials and Methods

### 2.1. Participants

The current sample consisted of 233 young adults from various universities in Singapore as part of a larger daily diary study exploring daily experiences and cognitive functions. Participants were included in the sample if they were undergraduates studying locally and signed up to take part in the study. They were considered nonclinical given that we did not measure if participants had been clinically diagnosed with borderline personality disorder. By recruiting a nonclinical sample, it allows the current study to obtain continuum data that include mild and extreme variants of borderline personality symptomatology [51]. Borderline personality symptomatology was measured by the 24-item Abbreviated Personality Assessment Inventory-Borderline Scale [52]. Specifically, borderline personality symptoms were manifested based on 4 subfactors of affective instability, identity problems, negative relationships, and self-harm, each of which consisted of 6 items. After the items are scored, higher scores on the PAI-BOR reflected greater presence of borderline personality disorder symptomatology in the participants.

Participants were recruited in waves from 1 July 2022 to 30 August 2022, and signed up for the study voluntarily. Participants were excluded from the sample if they were younger than age 18 or had participated in previous iterations of the daily diary study (which were carried out in 2021 and 2022) [53,54,55,56]. This study was conducted with the approval from the Institutional Review Board at the authors’ university, and participants provided informed consent to participate in the study in return for compensation of up to SGD 72. Descriptive statistics of the current sample can be found in Table 1. 

### 2.2. Measures

#### 2.2.1. Borderline Personality Disorder Symptomatology

In the current study, BPD was operationalised in terms of self-reported BPD symptomatology. BPD symptomatology was assessed in the sample using the 24-item Abbreviated Personality Assessment Inventory-Borderline Scale [52]. Participants were asked to rate their agreement with statements about their mood, attitudes, emotions, and behaviour on a 4-point Likert scale (0 = Not at all true, 3 = Very true). These items reflected BPD’s features of affective instability (6 items, e.g., “My mood can shift quite suddenly”), identity problems (6 items, e.g., “My attitude about myself changes a lot”), negative relationships (6 items, e.g., “My relationships have been stormy”), and self-harm (6 items, e.g., “I sometimes do things so impulsively that I get into trouble”). Items were scored and then summed such that a higher overall score reflected higher presence of BPD symptomatology in participants.

#### 2.2.2. Updating-Working Memory Capacity

Updating-working memory was assessed through a series of three tasks from Von Bastian et al. [58], modified from Miyake et al.’s [49] Keep-Track tasks, and administered on Tatool Web [59]. A total of 25 trials were carried out. In 5 of the trials, recall was probed early while in the other 20, recall was probed after multiple updating steps. Each participant’s accuracy across all trials served as a measure of their performance, which was obtained by dividing the number of trials correctly answered by the total number of trials (i.e., which was 25).

##### Colour Keep-Track Task 

This first task required participants to memorise an initial set of shapes and their respective colours, before needing participants to update this set as the colours changed. After updating, participants were asked to recall the most recent set of colours and pick the correct answer out of 10 options.

##### Letter Keep-Track Task 

This was the second task of the series and required participants to memorise specific letters, with each letter in its own box. One of the letters would be changed with each update, and after the full updating, participants again had to recall the most recent set before choosing the correct answer out of 10 options.

##### Number Keep-Track Task 

This was the last task of the series and required participants to memorise 4 digits with different colours. Each update would change the value of each digit and participants had to recall the most recent set after the full sequence of updates. Again, they would need to choose the correct answer out of the 10 presented options.

#### 2.2.3. Inhibitory Control

Inhibitory control was assessed through a series of three tasks administered on Tatool Web [58,59]. Participants had to respond to target stimuli shown on the screen and classify them while inhibiting distractor stimuli. Trials could be congruent, where the target stimuli’s category matched its distractor, or incongruent, where the stimuli were contradictory. In some cases, there were also neutral trials, where distractor stimuli were completely unrelated to the classification of target stimuli.

Participant’s inhibitory control was assessed using the binning procedure on each task [60], where higher bin scores reflected worse performance. First, a baseline score was calculated by averaging each participant’s total reaction time for their accurate congruent trials. This baseline represented participant’s natural response and ability. Next, the participant’s reaction time for each incongruent trial was compared with their baseline reaction time. The resulting difference is the interference effect, which represents how much slower the participants responded when they needed to inhibit the distractor stimuli. For each trial, all interference effects were rank ordered and bin scores were awarded based on deciles, where the fastest 10% would receive a score of 1 and the slowest 10% would receive a score of 10. To penalise for inaccuracy, any inaccurate incongruent response received a bin score of 20 regardless of reaction time. A mean bin score was then calculated for each participant.

##### Simon Task 

In this first task, participants had to respond to the colour of a circle presented on either side of the screen by pressing either the right (for a red circle) or left (for a green circle) arrow key. There were congruent, where the location of the circle on the screen matched the direction of the corresponding arrow keys (i.e., a green circle appearing on the left side of the screen, requiring participants to press the left arrow key), and incongruent trials where they did not match. The congruent arrangement accounted for 75% of the trials, and participant’s performance was assessed in conjunction with the two other inhibitory control tasks. There was a total of 200 trials, with 12 trials as practice. This task did not have neutral trials.

##### Erikson-Flanker Task 

In this second inhibitory control task, participants were presented with a central letter flanked by other letters and on each end was a vowel (A or E) or a consonant (S or T). They would then have to respond by pressing either the left arrow key for a vowel or the right arrow key for a consonant. The trials were either congruent, where vowels were flanked by vowels and vice versa for consonants (e.g., AAAEAAA or SSSTSSS), or incongruent, where the target letter was flanked by the opposite category (e.g., AAATAAA or SSSESSS). There were also neutral trials where the target letter was flanked by neutral stimuli (e.g., ###E###). These three arrangements occurred in equal proportions. There was a total of 144 trials, with 12 as practice.

##### Stroop Task 

This was the final task for inhibitory control and required participants to count the number of digits presented and respond by pressing the correct number key. While the task is commonly used to measure selective attention [61], extensive research has also established the use of the Stroop task as a measure of individual difference in inhibitory control [25,50,62,63,64]. There were congruent trials, where the number of digits corresponded to the digit displayed (e.g., 333) and incongruent trials, where the number of digits did not correspond to the digit displayed (e.g., 44). There were also neutral trials which presented unrelated symbols (e.g., ###). All three arrangements occurred in equal proportions. There was a total of 144 trials, with 12 as practice. This number-digit iteration was chosen over the traditional word-colour task given that it is relatively less well-known, which may reduce potential practice effect in the task. Moreover, the use of a numerical variant of the Stroop task is less susceptible to language proficiency and is more user friendly for individuals with different cultures and conditions [65]. The number-digit version has been validated and shown to elicit a robust Stroop Effect [66,67,68,69,70].

#### 2.2.4. Cognitive Flexibility

A set of three tasks were used to assess participant’s cognitive flexibility [58,59] and were administered on Tatool Web [59]. Participants were asked to classify bivalent stimuli according to the cue shown. These tasks were arranged in a sandwich-like order, where participants were given two single-rule blocks (20 trials per block), where they were only shown either animacy or locomotion stimuli, four mixed-rule blocks (41 trials per block), and finally ended off with two single-rule blocks (20 trials per block).

Participant’s cognitive flexibility was assessed using bin scores on each task [71,72,73], where higher bin scores reflected worse performance. First, a baseline score was calculated by averaging each participant’s total reaction time for their accurate single-rule (repeat) trials. This baseline represented participant’s natural response and ability. Next, participant’s reaction time for each mixed rule (switch) trial was compared with their baseline reaction time. The resulting difference is the switch cost, which represents how much slower the participants responded when they needed to switch between rules. For each trial, all switch costs were rank ordered and bin scores were awarded based on deciles, where the fastest 10% would receive a score of 1 and the slowest 10% would receive a score of 10. To penalise for inaccuracy, any inaccurate switch response received a bin score of 20 regardless of reaction time. A mean bin score was then calculated for each participant.

##### Animacy-Locomotion Task 

This first task required participants to classify the target stimuli of a plane and rabbit according to their animacy, whether the stimuli was animate or inanimate, or their locomotion, whether the stimuli was flying or non-flying. Animacy was cued with an image of dog paws and locomotion was cued by an image of a road and blue sky. When faced with the target stimuli, participants would have to either press the ‘d’ key if it was animate or flying or press the ‘k’ key if it was inanimate or non-flying.

##### Colour-Shape Task 

This second task required participants to classify the target stimuli of geometrical shapes in terms of their colour, either green or blue, or shape, either round or angular. Colour was cued using an image of a colour gradient while shape was cued using a row of small black diamonds. Participants would have to respond to the target stimuli and classify them accordingly by pressing the ‘d’ and ‘k’ keys for green or round and blue or angular, respectively.

##### Magnitude-Parity Task

This was the last task in the series and required participants to classify digits in terms of their parity, whether they were odd or even, and their magnitudes, whether they were greater or smaller than 5. Parity was cued with an image of rows of equal signs while magnitude was cued with an image of alternating columns of big and small circles. Participants responded to the target stimuli by pressing the ‘d’ and ‘k’ keys for odd or greater than 5 and even or smaller than 5, respectively.

### 2.3. Procedure

The study was carried out in three separate portions to minimise any fatigue. First, participants attended a 90 min session which consisted of a briefing, informed consent, and the cognitive measures. Participants were tested in a quiet setting to minimise distractions. They completed the nine executive function tasks in the following order: Colour Keep-Track, Letter Keep-Track, Number Keep-Track, Simon, Flanker, Stroop, Animacy-Locomotion, Colour-Shape, and Magnitude-Parity. Second, participants attended two baseline sessions, which totalled about 2 h, in a quiet setting where they responded to demographic questions in the first session, the PAI-BOR in the second session, and other questionnaires across the two sessions. Third, over the course of 7 days, participants filled in a daily diary with measures of affect and other questionnaires. The daily diary data were not analysed in the current paper.

### 2.4. Plan of Analysis

As the PAI-BOR has not been validated in an Asian sample before, the subfactors of affective instability, identity problems, negative relationships, and self-harm were estimated using confirmatory factor analysis. The four subfactors were manifested through 6 items each—items 1 (“My mood can shift quite suddenly”), 4 (“My moods get quite intense”), 7 (reverse coded “My mood is very steady”), 10 (“I have little control over my anger”), 14 (reverse -coded “I’ve always been a pretty happy person”) and 18 (“I’ve had times when I was so mad I couldn’t do enough to express all my anger”) were used to manifest affective instability; items 2 (“My attitude about myself changes a lot”), 5 (“Sometimes I feel terribly empty inside”), 8 (“I worry a lot about other people leaving me”), 11 (“I often wonder what I should do with my life”), 15 (“I can’t handle separation from those close to me very well”), and 19 (reverse-coded “I don’t get bored very easily”) were used to manifest identity problems; items 3 (“My relationships have been stormy”), 6 (“I want to let certain people know how much they’ve hurt me”), 9 (“People once close to me have let me down”), 12 (reverse-coded “I rarely feel very lonely”), 16 (“I’ve made some real mistakes in the people I’ve picked as friends”), and 20 (reverse-coded “Once someone is my friend, we stay friends”) were used to manifest negative relationships; and items 13 (“I sometimes do things so impulsively that I get into trouble”), 17 (“When I’m upset, I typically do something to hurt myself”), 21 (“I’m too impulsive for my own good”), 22 (“I spend money too easily”), 23 (“I’m a reckless person”), and 24 (reverse coded “I’m careful about how I spend my money”) were used to manifest self-harm.

The latent variables of updating-working memory, inhibitory control, and cognitive flexibility were first estimated using confirmatory factor analysis. The latent variables were manifested through the 9 tasks carried out during the cognitive sessions—Colour, Number, and Letter Keep-Track tasks were used to manifest updating-working memory capacity; Simon, Flanker, and Stroop tasks were used to manifest inhibitory control; and Animacy-Locomotion, Colour-Shape, and Magnitude-Parity tasks were used to manifest cognitive flexibility.

Subsequently, we ran a total of 10 unadjusted and adjusted structural equation models with each participant’s mean BPD scores as the outcome and executive functions as the predictor (as represented by the three latent variables). In the first model, we used the mean composite BPD symptom scores as the predictor, and this was regressed against the three executive function latent variables. In Models 2 to 5, we regressed the mean score of each BPD subfactor against the three executive function latent variables. Each adjusted model controlled for demographic covariates, namely, age, sex (dummy-coded with female = 1), race (dummy-coded with ethnic majority Chinese = 0), household income, and subjective socioeconomic status. This was undertaken to investigate if executive functions could significantly predict and account for BPD score outcomes, and the BPD score outcomes observed were not due to any demographic factors.

Several fit indices were used to check model fit, where better fit was indicated by a non-significant chi-square statistic χ^2^, higher values of Bentler’s comparative fit index (CFI) and the Tucker–Lewis index (TLI), and lower values of standardised root mean-squared residual (SRMR), and root mean square error of approximation (RMSEA). Although the chi-square statistic is oversensitive, it is still a widely used test of model fit, and as such we have included it. Established cut-offs were used to judge model fit, whereby an excellent model fit would be indicated by CFI ≈ 0.95, TLI ≈ 0.95, and SRMR ≈ 0.08 [74], as well as RMSEA < 0.06 [75]. Lower values of Akaike’s information criterion (AIC) and Bayesian information criterion (BIC) were used to compare between models.

### 2.5. Transparency and Openness

This study’s design and its analysis plan were not pre-registered. All data and code have been made publicly available on Researchbox (#930—https://researchbox.org/930). All analyses were conducted in R version 4.1.2 [76] using lavaan version 0.6–11 [77] set to mimic Mplus [78] in all calculations. We used full-information maximum likelihood parameter estimation to handle missing data.

## 3. Results

### 3.1. Confirmatory Factor Analysis

**Figure 1:**
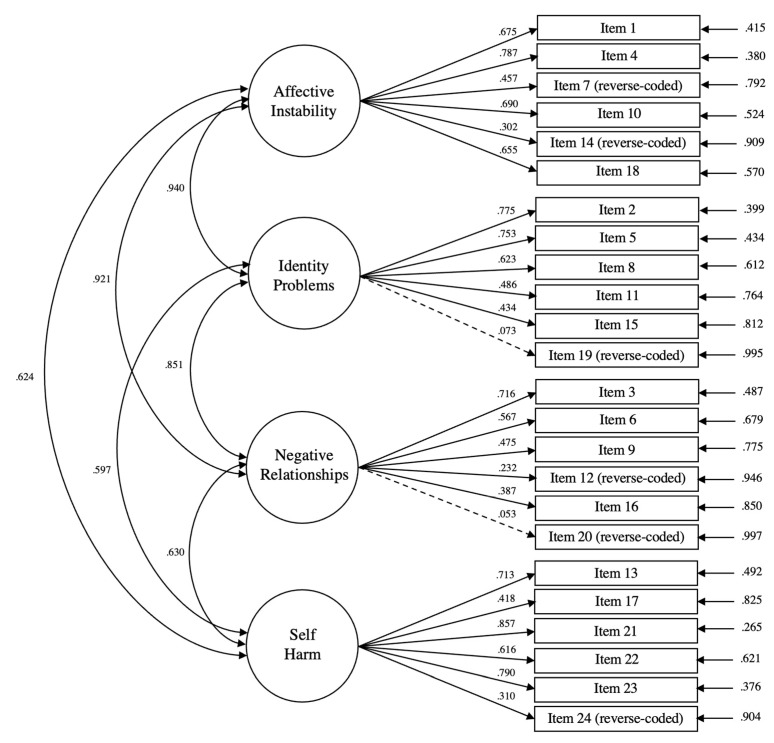
Confirmatory factor analysis of four-factor measurement model of BPD symptomatology. Circles represent latent variables while boxes represent manifest variables (tasks). Single-headed arrows connecting latent variables to manifest variables represent standardised factor loadings. Double-headed arrows connecting latent variables represent latent intercorrelations. Single-headed arrows on the right of the figure represent error terms. Solid lines represent significant relationships, while dashed lines represent non-significant relationships, at the 0.05 level.

The fit of the four-factor measurement model of BPD symptomatology was acceptable, CFI = 0.776, TLI = 0.746, SRMR = 0.087, RMSEA = 0.094, and most of the manifest variables loaded significantly onto their respective latent subfactors of interest. While the chi-square test was statistically significant (*p* < 0.001), suggesting that the model was significantly different from data, this was to be expected as the chi-square test statistic is oversensitive to large sample sizes [79,80]. Two manifest variables (item 19 “I don’t get bored very easily” and item 20 “Once someone is my friend, we stay friends”) did not load significantly onto their corresponding subfactors of identity problems and negative relationships. In accordance with previous works, there were significant latent intercorrelations between the four subfactors of affective instability, identity problems, negative relationships, and self-harm (*r*s = [0.233, 0.445], all *p*s < 0.001). Figure 1 depicts the confirmatory factor analysis used to assess the measurement model.

**Figure 2:**
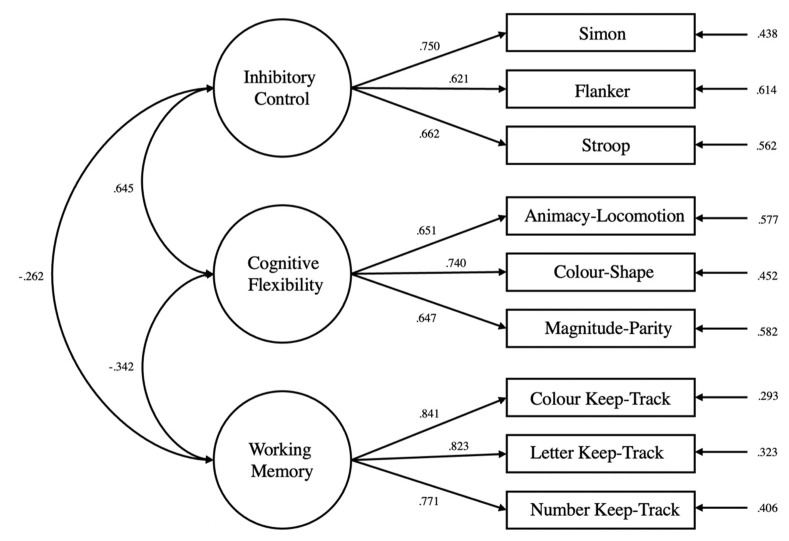
Confirmatory factor analysis of three-factor model of executive functions. Circles represent latent variables while boxes represent manifest variables (tasks). Single-headed arrows connecting latent variables to manifest variables represent standardised factor loadings. Double-headed arrows connecting latent variables represent latent intercorrelations. Single-headed arrows on the right of the figure represent error terms. All correlations and loadings were significant at the 0.05 level.

Consistent with the three-factor model of executive functions hypothesised by Miyake, Friedman et al. (2000), the fit of the current three-factor model was excellent: CFI = 0.977, TLI = 0.966, SRMR = 0.047, RMSEA = 0.051. Similar to previous works, each manifest variable loaded significantly onto its latent variable of interest (*p*s < 0.001), and there were significant latent correlations between inhibitory control and task switching (*r* = 0.635, *p* < 0.001), between inhibitory control and updating-working memory (*r* = −0.049, *p* = 0.004), and between task switching and updating-working memory (*r* = −0.038, *p* < 0.001). Figure 2 depicts the confirmatory factor analysis used to assess the measurement model.

### 3.2. Structural Equation Modelling

The models showed excellent fits as seen from the summary statistics in Table 2. Table 3 summarises the standardised coefficient estimates of the three latent variables of updating-working memory capacity, inhibitory control, and task-switching for the composite BPD score and four subfactors of affective instability, identity problems, negative relationships, and self-harm in adjusted and unadjusted Models 1 to 5.

**Figure 3:**
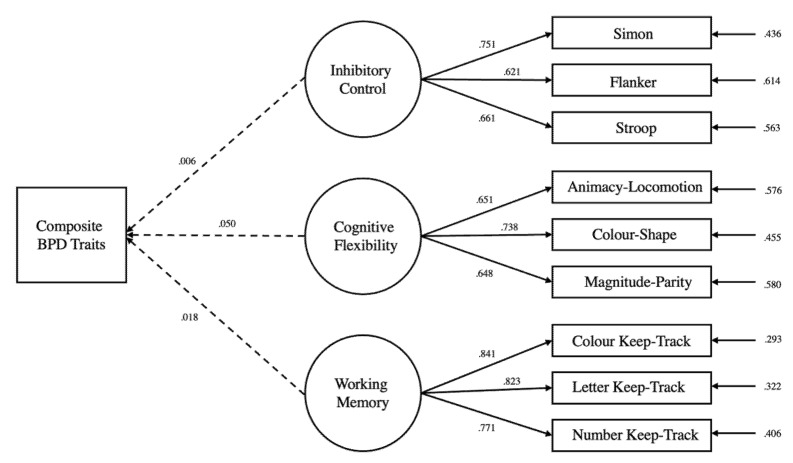
Structural equation model of the composite BPD regressed against three latent variable executive function. Circles represent latent variables while boxes represent manifest variables. Single-headed arrows connecting latent variables to manifest variables represent standardised factor loadings. Single-headed arrows connecting executive function latent variables to the composite BPD traits represent regressions. Single-headed arrows on the right of the figure represent error terms. The intercorrelations between the executive functions were modelled but not displayed in the figure. Solid lines represent significant relationships, while dashed line represents non-significant relationships, at the 0.05 level.

**Figure 4:**
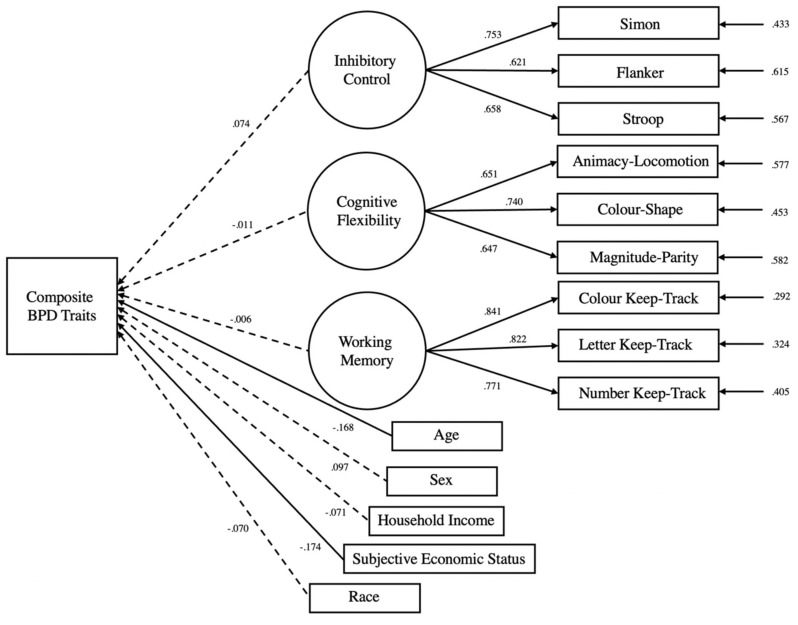
Structural equation model of the composite BPD regressed against three latent variable executive function and covariates. Circles represent latent variables while boxes represent manifest variables. Single-headed arrows connecting latent variables to manifest variables represent standardised factor loadings. Single-headed arrows connecting executive function latent variables to the composite BPD traits represent regressions. Single-headed arrows on the right of the figure represent error terms. The intercorrelations between the executive functions were modelled but not displayed in the figure. Solid lines represent significant relationships, while dashed line represents non-significant relationships, at the 0.05 level.

Executive functions were not significantly associated with BPD symptomatology across all models. Inhibitory control was not significantly associated with the composite BPD score (unadjusted: β = 0.006, *z* = 0.04, *p =* 0.963; adjusted: β = 0.074, *z* = 0.64, *p =* 0.517), affective instability (unadjusted: β = −0.079, *z* = −0.66, *p* = 0.509; adjusted: β = −0.002, *z* = −0.02, *p =* 0.983), identity problems (unadjusted: β = −0.012, *z* = −0.09, *p* = 0.923; adjusted: β = 0.062, *z* = 0.54, *p* = 0.589), negative relationships (unadjusted: β = −0.054, *z* = −0.454, *p* = 0.650; adjusted: β = −0.019, *z* = −0.16, *p* = 0.870), and self-harm (unadjusted: β = 0.155, *z* = 1.29, *p =* 0.194; adjusted: β = 0.189, *z* = 1.59, *p* = 0.111). Similarly, cognitive flexibility was not significantly associated with the composite BPD score (unadjusted: β = 0.050, *z* = 0.40, *p* = 0.687; adjusted: β = −0.011, *z* = −0.09, *p =* 0.927), affective instability (unadjusted: β = 0.148, *z* = 1.193, *p =* 0.233; adjusted: β = 0.082, *z* = 0.686, *p =* 0.493), identity problems (unadjusted: β = 0.036, *z* = 0.29, *p* = 0.923; adjusted: β = −0.036, *z* = −0.304, *p =* 0.761), negative relationships (unadjusted: β = 0.120, *z* = 0.96, *p =* 0.336; adjusted: β = 0.101, *z* = 0.08, *p =* 0.414), and self-harm (unadjusted: β = −0.133, *z* = −1.07, *p =* 0.284; adjusted: β = −0.167, *z* = −1.34, *p =* 0.178). Likewise, updating-working memory was not significantly associated with the composite BPD score (unadjusted: β = 0.018, *z* = 0.23, *p =* 0.817; adjusted: β = −0.006, *z* = −0.07, *p =* 0.938), affective instability (unadjusted: β = 0.017, *z* = 0.21, *p =* 0.827; adjusted: β = 0.001, *z* = 0.00, *p =* 0.993), identity problems (unadjusted: β = 0.097, *z* = 1.24, *p =* 0.213; adjusted: β = 0.054, *z* = 0.71, *p =* 0.475), negative relationships (unadjusted: β = 0.004, *z* = 0.055, *p =* 0.956; adjusted: β = 0.011, *z* = 0.14, *p =* 0.888), and self-harm (unadjusted: β = −0.059, *z* = −0.75, *p =* 0.444; adjusted: β = −0.081, *z* = −1.04, *p =* 0.299). Some significant associations were however found between the executive functions and the covariates in the adjusted models, as seen from the highlighted coefficients in Table 3. Figure 3 depicts the structural equation modelling employed in the unadjusted composite BPD model, while Figure 4 depicts the structural equation modelling employed in the adjusted composite BPD model. The figures for the unadjusted and unadjusted individual BPD subfactor models can be found in the supplement Researchbox (#930).

## 4. Discussion

The current study aimed to expand the existing literature on the underlying cognitive mechanisms in BPD symptomatology by exploring the relationship between executive functions and BPD symptomatology in a nonclinical population. Based on the results of our structural equation modelling in all unadjusted and adjusted models, we did not find strong evidence that executive functions, in the domains of inhibitory control, cognitive flexibility, and updating-working memory, were associated with the four subfactors of BPD symptomatology—namely, affective instability, identity problems, negative relationships, and self-harm—nor with overall BPD symptomatology in a nonclinical population.

Our novel study showed no evidence that suggests deficits in executive function can be a risk factor for the manifestation of BPD symptomatology in nonclinical populations. While executive functions have been shown to predict many important life outcomes [26], our study identifies an important boundary condition of the role of executive functions in BPD symptomatology. It is plausible that BPD symptomatology in nonclinical populations is less cognitive in nature. The lack of association between the latent factor of executive functions and BPD symptomatology could also be driven by the distinction between BPD symptomatology between clinical and nonclinical populations. For instance, BPD symptomatology in nonclinical populations tends to be less severe and more context-dependent than BPD symptomatology in clinical populations [16,81]. Thus, situational factors such as exposure to stressors and relational quality [82,83,84,85,86,87] may predict BPD symptomatology in nonclinical populations better than stable cognitive traits such as executive functions [88,89]. Alternatively, another possibility for the lack of evidence found in the current study could be that executive functions peak between late adolescence to adulthood, between the ages of 20 to 29 [90,91,92,93,94]. Our sample’s observed age range was between 18 to 30, which matches the proposed peak executive function age band. Thus, there might be more range restrictions in the executive functioning of young adults that may attenuate the link between executive functions and BPD symptomatology.

However, it should be noted that these results should not discourage the employment of effective treatments for executive functioning, and its associated domains of behavioural impulsivity, emotional reactivity, and emotional dysregulation, in clinical populations diagnosed with BPD. These treatments, consisting of cognitive remediation [95,96] and brain stimulation [97,98], have been shown to have benefits in enhancing and alleviating typical BPD symptomatology that should not be overlooked.

It is noteworthy that the current study addressed several prominent methodological and statistical issues in the field. Firstly, our study recognised the multidimensional aspect of executive functions and encapsulated this through the battery of nine tasks in the three executive function domains (inhibitory control, cognitive flexibility, and updating-working memory). Thus, we can rule out the possibility that the null results found in the current study are simply due to the possibility that the relations between executive functions and BPD symptomatology in nonclinical populations are domain-specific. Secondly, the present research leveraged on the use of robust latent variable analysis to address the issue of task impurity in the executive function tasks. This reduces type II error rates through increased statistical power, while also reducing type I error rates [64].

Our findings do however have their limitations. Firstly, the present study is correlational in nature and is thus limited in its interpretability of directionality. While our study used the logic that poor executive functions result in greater BPD symptomatology, it is entirely possible that greater BPD symptomatology results in poorer executive functions. Furthermore, the present study is cross-sectional in nature, using only one time point of executive functioning and BPD symptomatology data. Both executive functions [90] and BPD symptomatology [99] have been found to fluctuate over time as an individual ages, so future studies could thus utilise longitudinal and experimental methods to further examine the directionality and stability of the relationship between executive functions and BPD symptomatology. The current study also utilised the PAI-BOR scale, which has not been validated in Asian samples. As such, it could be possible that the items might not accurately represent or reflect subfactors present in the Asian demographic. Future studies could thus test the validity of the PAI-BOR in Asian samples.

In sum, the current study shows that deficits in executive functions may not be a risk factor for the manifestation of BPD symptomatology in young adults. The finding has given critical insights towards the relatively unexplored association between executive functions and BPD traits. Given that BPD symptomatology develops differently between clinical and nonclinical populations, the findings bolster the need to investigate this disparity further.

## Figures and Tables

**Table 1:** Descriptive statistics of the sample.

	*N*	*M* (*SD*)	Observed Range	Theoretical Range	Reliability ^d^
**Demographics**					
Sex (% female)	233	73%			
Age	233	21.64 (2.00)	18–30		
Race (% Ethnic Majority)	233	79%			
Household income ^a^	233	3.15 (1.57)	1–6	1–6	
Subjective socioeconomic status ^b^	233	6.10 (1.35)	2–9	1–10	
**Borderline Personality Disorder Trait Dimensions**					
Affective Instability	233	7.03 (3.52)	0–18	0–18	0.77
Identity Problems	233	9.14 (3.64)	1–18	0–18	0.69
Negative Relationships	233	8.17 (3.23)	1–17	0–18	0.53
Self-Harm	233	5.11 (3.73)	0–18	0–18	0.79
**Executive functions ^c^**					
Colour Keep-Track	233	0.56 (0.17)	0.09–0.94	0.00–1.00	0.95
Letter Keep-Track	233	0.72 (0.14)	0.07–0.99	0.00–1.00	0.94
Number Keep-Track	233	0.71 (0.21)	0.16–1.00	0.00–1.00	0.96
Simon task	232	7.12 (1.71)	3.28–12.04	1–20	0.79
Flanker task	231	6.43 (1.31)	3.72–14.89	1–20	0.72
Stroop task	227	6.78 (1.38)	3.49–11.55	1–20	0.79
Animacy-Size task	233	6.74 (1.18)	4.38–13.57	1–20	0.73
Colour-Shape task	233	6.90 (1.30)	4.36–13.00	1–20	0.72
Magnitude-Parity task	233	7.13 (1.40)	3.23–11.93	1–20	0.75

^a^ Household income was measured using a 6-point scale (1 = less than SGD 2000, 2 = SGD 2000–SGD 5999, 3 = SGD 6000–SGD 9999, 4 = SGD 10,000–SGD 14,999, 5 = SGD 15,000–SGD 19,999, 6 = more than SGD 20,000). ^b^ A modified ladder scale adapted from Adler et al. [57] was used to assess subjective socioeconomic status, where the first rung on the ladder represented the lowest status. ^c^ Some data were missing due to technical failures and human error by the participants. ^d^ Internal consistency was measured with Cronbach’s Alpha for the BPD scale and via split-half reliability with Spearman–Brown correction for the executive function tasks.

**Table 2:** Summary of model fits.

	*df*	χ^2^	pχ^2^	AIC	BIC	SRMR	RMSEA	CFI	TLI
**Confirmatory Factor Analysis**									
PAI-BOR	246	750.16	0.000	13610.63	13879.81	0.081	0.094	0.758	0.729
Executive Functions	24	38.780	0.029	3748.63	3852.16	0.047	0.051	0.977	0.966
**Structural Equation Modelling**									
**Unadjusted**									
Composite BPD	30	46.082	0.031	4079.27	4200.06	0.045	0.048	0.975	0.963
Affective Instability	30	49.680	0.013	4198.45	4319.24	0.047	0.053	0.970	0.955
Identity Problem	30	47.560	0.022	4184.18	4304.97	0.046	0.050	0.973	0.960
Negative Relationships	30	45.407	0.035	4128.72	4249.50	0.045	0.047	0.976	0.964
Self-harm	30	43.834	0.049	4195.54	4316.33	0.045	0.044	0.979	0.968
**Adjusted**									
Composite BPD	75	118.82	0.001	4067.36	4205.40	0.056	0.050	0.937	0.920
Affective Instability	75	121.52	0.001	4184.35	4322.39	0.056	0.052	0.934	0.916
Identity Problem	75	119.70	0.001	4169.24	4307.28	0.056	0.051	0.936	0.919
Negative Relationships	75	118.57	0.001	4129.56	4267.60	0.056	0.050	0.936	0.919
Self-harm	75	118.09	0.001	4196.69	4334.73	0.056	0.050	0.937	0.920

Note. AIC = Akaike’s information criterion; BIC = Bayesian information criterion; SRMR = standardised root mean-squared residual; RMSEA = root mean square error of approximation; CFI = Bentler’s comparative fit index; TLI = Tucker–Lewis index. Lower values of AIC, BIC, SRMR, and RMSEA indicate better fit. Higher values of CFI and TLI indicate better fit. Unadjusted models include the composite BPD trait score as well as individual latent subfactors, while the adjusted models additionally include the covariates of age, sex, race, household income, and subjective socioeconomic status.

**Table 3:** Standardised coefficient estimates of three latent variables of inhibitory control, cognitive flexibility, and updating-working memory capacity predicting composite BPD and four subfactors of affective instability, identity problems, negative relationships, and self-harm.

	Composite BPD		Affective Instability		Identity Problems		Negative Relationships		Self-Harm	
Predictor	Unadjusted	Adjusted	Unadjusted	Adjusted	Unadjusted	Adjusted	Unadjusted	Adjusted	Unadjusted	**Adjusted**
**Executive Functions**										
Inhibitory control	0.006	0.074	−0.079	−0.002	−0.012	0.062	−0.054	−0.019	0.155	0.189
Cognitive flexibility	0.050	−0.011	0.148	0.082	0.036	−0.036	0.120	0.101	−0.133	−0.167
Working memory	0.018	−0.006	0.017	0.001	0.097	0.054	0.004	0.011	−0.059	−0.081
**Covariates**										
Age		−0.168 *		−0.120		−0.172 *		−0.070		−0.174 *
Sex (0 = male, 1 = female)		0.097		0.157*		0.131		0.013		0.005
Household income		−0.071		−0.080		−0.052		−0.089		−0.013
Subjective socioeconomic status		−0.174 *		−0.180 *		−0.155 *		−0.136		−0.090
Race (0 = majority, 1 = minority)		−0.070		−0.042		−0.130 *		−0.018		−0.032

Note. *N* = 233. * Regressions were statistically significant at *p* < 0.05. The rest of the regressions were non-significant.

## Data Availability

The dataset can be downloaded at https://researchbox.org/930.

## References

[1] Paris J. (2005). Borderline personality disorder. Can. Med Assoc. J..

[2] Cleary M., Siegfried N., Walter G. (2002). Experience, knowledge and attitudes of mental health staff regarding clients with a borderline personality disorder. Int. J. Ment. Health Nurs..

[3] Sulzer S.H. (2015). Does “difficult patient” status contribute to de facto demedicalization? The case of borderline personality disorder. Soc. Sci. Med..

[4] Allen A., Links P.S. (2011). Aggression in Borderline Personality Disorder: Evidence for Increased Risk and Clinical Predictors. Curr. Psychiatry Rep..

[5] IsHak W.W., Elbau I., Ismail A., Delaloye S., Ha K., Bolotaulo N.I., Nashawati R., Cassmassi B., Wang C. (2013). Quality of Life in Borderline Personality Disorder. Harv. Rev. Psychiatry.

[6] American Psychiatric Association (2013). Diagnostic and Statistical Manual of Mental Disorders.

[7] Sayrs J., Whiteside U., Fisher J.E., O’Donohue W.T. (2006). Borderline Personality Disorder. Practitioner’s Guide to Evidence-Based Psychotherapy.

[8] Aviram R.B., Brodsky B.S., Stanley B. (2006). Borderline Personality Disorder, Stigma, and Treatment Implications. Harv. Rev. Psychiatry.

[9] Coolidge F.L., Segal D.L., Stewart S.E., Ellett J.A. (2000). Neuropsychological Dysfunction in Children with Borderline Personality Disorder Features: A Preliminary Investigation. J. Res. Pers..

[10] Jenkins C.A., Thompson K.N., Nicholas C.L., Hartmann J.A., Chanen A.M. (2022). Potential mechanisms underlying sleep disturbance in young people with borderline personality disorder features: An exploratory study. Bord. Pers. Disord. Emot. Dysregulation.

[11] Paris J. (2019). Suicidality in Borderline Personality Disorder. Medicina.

[12] Paris J., Zweig-Frank H. (2001). A 27-year follow-up of patients with borderline personality disorder. Compr. Psychiatry.

[13] Shen C., Wang M.P., Chu J.T., Wan A., Viswanath K., Chan S.S.C., Lam T.H., Bacigalupe G., Zhang M., Sapkota B. (2017). Sharing Family Life Information Through Video Calls and Other Information and Communication Technologies and the Association With Family Well-Being: Population-Based Survey. JMIR Ment. Health.

[14] Lieb K., Zanarini M.C., Schmahl C., Linehan M.M., Bohus M. (2004). Borderline personality disorder. Lancet.

[15] Stone M.H., Friedman H.J. (1990). The Fate of Borderline Patients: Successful Outcome and Psychiatric Practice.

[16] Trull T.J. (1995). Borderline personality disorder features in nonclinical young adults: 1. Identification and validation. Psychol. Assess..

[17] Ellison W.D., Rosenstein L.K., Morgan T.A., Zimmerman M. (2018). Community and Clinical Epidemiology of Borderline Personality Disorder. Psychiatr. Clin. North Am..

[18] Winsper C., Zanarini M., Wolke D. (2012). Prospective study of family adversity and maladaptive parenting in childhood and borderline personality disorder symptoms in a non-clinical population at 11 years. Psychol. Med..

[19] Fonseca-Pedrero E., Paino M., Lemos-Giráldez S., Sierra-Baigrie S., González M.P.G.-P., Bobes J., Muňiz J. (2011). Borderline Personality Traits in Nonclinical Young Adults. J. Pers. Disord..

[20] Daley S.E., Burge D., Hammen C. (2000). Borderline personality disorder symptoms as predictors of 4-year romantic relationship dysfunction in young women: Addressing issues of specificity. J. Abnorm. Psychol..

[21] Vega D., Torrubia R., Marco-Pallarés J., Soto A., Rodriguez-Fornells A. (2020). Metacognition of daily self-regulation processes and personality traits in borderline personality disorder. J. Affect. Disord..

[22] Németh N., Péterfalvi Á., Czéh B., Tényi T., Simon M. (2020). Examining the Relationship Between Executive Functions and Mentalizing Abilities of Patients With Borderline Personality Disorder. Front. Psychol..

[23] McClure G., Hawes D.J., Dadds M. (2015). Borderline personality disorder and neuropsychological measures of executive function: A systematic review. Pers. Ment. Health.

[24] Wright A.G.C., Hallquist M.N., Stepp S., Scott L.N., Beeney J.E., Lazarus S.A., Pilkonis P.A. (2016). Modeling Heterogeneity in Momentary Interpersonal and Affective Dynamic Processes in Borderline Personality Disorder. Assessment.

[25] Miyake A., Friedman N.P., Emerson M.J., Witzki A.H., Howerter A., Wager T.D. (2000). The Unity and Diversity of Executive Functions and Their Contributions to Complex “Frontal Lobe” Tasks: A Latent Variable Analysis. Cogn. Psychol..

[26] Diamond A. (2013). Executive Functions. Annu. Rev. Psychol..

[27] Frontiers | A Bidirectional Relationship between Executive Function and Health Behavior: Evidence, Implica-tions, and Future Directions. https://www.frontiersin.org/articles/10.3389/fnins.2016.00386/full.

[28] Ng W.Q., Hartanto A. (2022). The effect of executive function on the development of chronic pain: A prospective longitudinal study. Soc. Sci. Med..

[29] Hartanto A., Yong J.C., Toh W.X. (2019). Bidirectional Associations between Obesity and Cognitive Function in Midlife Adults: A Longitudinal Study. Nutrients.

[30] Johnson J.K., Lui L.-Y., Yaffe K. (2007). Executive Function, More Than Global Cognition, Predicts Functional Decline and Mortality in Elderly Women. J. Gerontol. Ser. A.

[31] St Clair-Thompson H.L., Gathercole S.E. (2006). Executive functions and achievements in school: Shifting, updating, inhibition, and working memory. Q. J. Exp. Psychol..

[32] Titz C., Karbach J. (2014). Working memory and executive functions: Effects of training on academic achievement. Psychol. Res..

[33] Hartanto A., Yang H., Yang S. (2018). Bilingualism positively predicts mathematical competence: Evidence from two large-scale studies. Learn. Individ. Differ..

[34] Hartanto A., Yang H. (2022). Testing theoretical assumptions underlying the relation between anxiety, mind wandering, and task-switching: A diffusion model analysis. Emotion.

[35] Pe M.L., Koval P., Kuppens P. (2013). Executive well-being: Updating of positive stimuli in working memory is associated with subjective well-being. Cognition.

[36] Tan C.-S., Nasir H., Pheh K.-S., Cong C.W., Tay K.-W., Cheong J.-Q. (2022). The Mediating Role of Work Engagement in the Relationship between Executive Functioning Deficits and Employee Well-Being. Int. J. Environ. Res. Public Health.

[37] Toh W.X., Yang H., Hartanto A. (2019). Executive Function and Subjective Well-being in Middle and Late Adulthood. J. Gerontol. Ser. B.

[38] Moriguchi Y. (2014). The early development of executive function and its relation to social interaction: A brief review. Front. Psychol..

[39] Riggs N.R., Jahromi L.B., Razza R.P., Dillworth-Bart J.E., Mueller U. (2006). Executive function and the promotion of social–emotional competence. J. Appl. Dev. Psychol..

[40] Cropley M., Collis H. (2020). The Association Between Work-Related Rumination and Executive Function Using the Behavior Rating Inventory of Executive Function. Front. Psychol..

[41] Fisher G.G., Chaffee D.S., Tetrick L.E., Davalos D.B., Potter G.G. (2017). Cognitive functioning, aging, and work: A review and recommendations for research and practice. J. Occup. Health Psychol..

[42] Collins A., Koechlin E. (2012). Reasoning, Learning, and Creativity: Frontal Lobe Function and Human Decision-Making. PLOS Biol..

[43] Hagenhoff M., Franzen N., Koppe G., Baer N., Scheibel N., Sammer G., Gallhofer B., Lis S. (2013). Executive functions in borderline personality disorder. Psychiatry Res..

[44] Putnam K.M., Silk K.R. (2005). Emotion dysregulation and the development of borderline personality disorder. Dev. Psychopathol..

[45] Bickel W.K., Jarmolowicz D.P., Mueller E.T., Gatchalian K.M., McClure S.M. (2012). Are executive function and impulsivity antipodes? A conceptual reconstruction with special reference to addiction. Psychopharmacology.

[46] Hendrawan D., Yamakawa K., Kimura M., Murakami H., Ohira H. (2012). Executive functioning performance predicts subjective and physiological acute stress reactivity: Preliminary results. Int. J. Psychophysiol..

[47] Lantrip C., Isquith P.K., Koven N.S., Welsh K., Roth R.M. (2014). Executive Function and Emotion Regulation Strategy Use in Adolescents. Appl. Neuropsychol. Child.

[48] Marceau E.M., Kelly P.J., Solowij N. (2018). The relationship between executive functions and emotion regulation in females attending therapeutic community treatment for substance use disorder. Drug Alcohol Depend..

[49] Miyake A., Emerson M.J., Friedman N.P. (2000). Assessment of executive functions in clinical settings: Problems and recommendations. Semin. Speech Lang..

[50] Friedman N.P., Miyake A. (2004). The Relations Among Inhibition and Interference Control Functions: A Latent-Variable Analysis. J. Exp. Psychol. Gen..

[51] Carr S., Francis A. (2009). Childhood maltreatment and adult personality disorder symptoms in a non-clinical sample. Aust. Psychol..

[52] Morey L.C. (1991). Personality Assessment Inventory: Professional Manual.

[53] Hartanto A., Lee K.Y.X., Chua Y.J., Quek F.Y.X., Majeed N.M. (2022). Smartphone use and daily cognitive failures: A critical examination using a daily diary approach with objective smartphone measures. Br. J. Psychol..

[54] Ng M.H., Lua V.Y., Majeed N.M., Hartanto A. (2022). Does trait self-esteem serve as a protective factor in maintaining daily affective well-being? Multilevel analyses of daily diary studies in the US and Singapore. Pers. Individ. Differ..

[55] Lua V.Y.Q., Majeed N.M., Leung A.K.-Y., Hartanto A. (2022). A daily within-person investigation on the link between social expectancies to be busy and emotional wellbeing: The moderating role of emotional complexity acceptance. Cogn. Emot..

[56] Hartanto A., Wong J., Lua V.Y.Q., Tng G.Y.Q., Kasturiratna K.T.A.S., Majeed N.M. (2022). A Daily Diary Investigation of the Fear of Missing Out and Diminishing Daily Emotional Well-Being: The Moderating Role of Cognitive Reappraisal. Psychol. Rep..

[57] Adler N.E., Epel E.S., Castellazzo G., Ickovics J.R. (2000). Relationship of Subjective and Objective Social Status with Psychological and Physiological Functioning: Preliminary Data in Healthy, White Women. Health Psychol..

[58] von Bastian C.C., Souza A.S., Gade M. (2016). No evidence for bilingual cognitive advantages: A test of four hypotheses. J. Exp. Psychol. Gen..

[59] Von Bastian C.C., Locher A., Ruflin M. (2012). Tatool: A Java-based open-source programming framework for psychological studies. Behav. Res. Methods.

[60] Draheim C., Hicks K.L., Engle R.W. (2016). Combining Reaction Time and Accuracy. Perspect. Psychol. Sci..

[61] Simeonova D., Paunova R., Stoyanova K., Todeva-Radneva A., Kandilarova S., Stoyanov D. (2022). Functional MRI Correlates of Stroop N-Back Test Underpin the Diagnosis of Major Depression. J. Integr. Neurosci..

[62] Gärtner A., Strobel A. (2021). Individual Differences in Inhibitory Control: A latent Variable Analysis. J. Cogn..

[63] West R., Alain C. (2000). Age-related decline in inhibitory control contributes to the increased Stroop effect observed in older adults. Psychophysiology.

[64] Friedman N.P. (2016). Research on individual differences in executive functions. Represent. Process. Biling. Morphol..

[65] Lang J.A. (2002). Validation of the Five Digit Test in a Clinical Sample: An Alternative to the Stroop Color-Word Task with Possible Cultural Implications. Ph.D. Thesis.

[66] Wolach A.H., McHale M.A., Tarlea A. (2004). Numerical Stroop Effect. Percept. Mot. Ski..

[67] Bush G., Whalen P.J., Rosen B.R., Jenike M.A., McInerney S.C., Rauch S.L. (1998). The counting stroop: An in-terference task specialized for functional neuroimaging-validation study with functional MRI. Hum. Brain Mapp..

[68] Bush G., Whalen P.J., Shin L.M., Rauch S.L. (2006). The counting Stroop: A cognitive interference task. Nat. Protoc..

[69] Szűcs D., Soltész F. (2007). Event-related potentials dissociate facilitation and interference effects in the numerical Stroop paradigm. Neuropsychologia.

[70] Kaufmann L., Ischebeck A., Weiss E., Koppelstaetter F., Siedentopf C., Vogel S.E., Gotwald T., Marksteiner J., Wood G. (2008). An fMRI study of the numerical Stroop task in individuals with and without minimal cognitive impairment. Cortex.

[71] Hartanto A., Yang H. (2020). The role of bilingual interactional contexts in predicting interindividual variability in executive functions: A latent variable analysis. J. Exp. Psychol. Gen..

[72] Hartanto A., Ong N.C.H., Ng W.Q., Majeed N.M. (2020). The Effect of State Gratitude on Cognitive Flexibility: A Within-Subject Experimental Approach. Brain Sci..

[73] Hughes J.N., Im M.H., Wehrly S.E. (2014). Effect of peer nominations of teacher–student support at individual and classroom levels on social and academic outcomes. J. Sch. Psychol..

[74] Hu L.T., Bentler P.M. (1999). Cutoff criteria for fit indexes in covariance structure analysis: Conventional criteria versus new alternatives. Struct. Equ. Model. Multidiscip. J..

[75] Browne M.W., Cudeck R. (1992). Alternative ways of assessing model fit. Sociol. Methods Res..

[76] R Core Team (2022). R: A Language and Environment for Statistical Computing.

[77] Rosseel Y. (2012). lavaan: AnRPackage for Structural Equation Modeling. J. Stat. Softw..

[78] Muthén L.K., Muthén B.O. (2012). Mplus User’s Guide.

[79] Bergh D., Zhang Q., Yang H. (2015). Sample Size and Chi-Squared Test of Fit—A Comparison Between a Random Sample Approach and a Chi-Square Value Adjustment Method Using Swedish Adolescent Data. Pacific Rim Objective Measurement Symposium (PROMS) 2014 Conference Proceedings.

[80] Martin-Löf P. (1974). The Notion of Redundancy and Its Use as a Quantitative Measure of the Discrepancy between a Statistical Hypothesis and a Set of Observational Data [with Discussion]. Scand. J. Stat..

[81] Tyrer P., Reed G.M., Crawford M.J. (2015). Classification, assessment, prevalence, and effect of personality disorder. Lancet.

[82] Bradley G.L., Sparks B.A., Weber K. (2016). Perceived prevalence and personal impact of negative online reviews. J. Serv. Manag..

[83] Weaver T.L., Clum G.A. (1993). Early family environments and traumatic experiences associated with borderline personality disorder. J. Consult. Clin. Psychol..

[84] Bourvis N., Aouidad A., Cabelguen C., Cohen D., Xavier J. (2017). How Do Stress Exposure and Stress Regulation Relate to Borderline Personality Disorder?. Front. Psychol..

[85] Zielinski M.J., Veilleux J.C. (2014). Examining the relation between borderline personality features and social support: The mediating role of rejection sensitivity. Pers. Individ. Differ..

[86] Lavner J.A., Lamkin J., Miller J.D. (2015). Borderline personality disorder symptoms and newlyweds’ observed communication, partner characteristics, and longitudinal marital outcomes. J. Abnorm. Psychol..

[87] Levy K.N. (2005). The implications of attachment theory and research for understanding borderline personality disorder. Dev. Psychopathol..

[88] Friedman N.P., Miyake A., Young S.E., DeFries J.C., Corley R.P., Hewitt J.K. (2008). Individual differences in executive functions are almost entirely genetic in origin. J. Exp. Psychol. Gen..

[89] Logue S.F., Gould T.J. (2014). The neural and genetic basis of executive function: Attention, cognitive flexibility, and response inhibition. Pharmacol. Biochem. Behav..

[90] Ferguson H.J., Brunsdon V.E.A., Bradford E.E.F. (2021). The developmental trajectories of executive function from adolescence to old age. Sci. Rep..

[91] Friedman N.P., Miyake A. (2017). Unity and diversity of executive functions: Individual differences as a window on cognitive structure. Cortex.

[92] Luciana M., Conklin H.M., Hooper C.J., Yarger R.S. (2005). The Development of Nonverbal Working Memory and Executive Control Processes in Adolescents. Child Dev..

[93] Hartanto A., Toh W.X., Yang H. (2016). Age matters: The effect of onset age of video game play on task-switching abilities. Atten. Percept. Psychophys.

[94] Åberg M.A.I., Pedersen N.L., Torén K., Svartengren M., Bäckstrand B., Johnsson T., Cooper-Kuhn C.M., Åberg N.D., Nilsson M., Kuhn H.G. (2009). Cardiovascular fitness is associated with cognition in young adulthood. Proc. Natl. Acad. Sci. USA.

[95] Pascual J.C., Palomares N., Ibáñez Á., Portella M.J., Arza R., Reyes R., Feliu-Soler A., Díaz-Marsá M., Saiz-Ruiz J., Soler J. (2015). Efficacy of cognitive rehabilitation on psychosocial functioning in Borderline Personality Disorder: A randomized controlled trial. BMC Psychiatry.

[96] Vita A., Deste G., Barlati S., Poli R., Cacciani P., De Peri L., Sacchetti E. (2016). Feasibility and effectiveness of cognitive remediation in the treatment of borderline personality disorder. Neuropsychol. Rehabil..

[97] Chiappini S., Picutti E., Alessi M.C., Di Carlo F., D’Andrea G., Miuli A., Pettorruso M., Martinotti G., di Giannantonio M. (2022). Efficacy of Noninvasive Brain Stimulation on Borderline Personality Disorder Core Symptoms: A Systematic Review. J. Pers. Disord..

[98] Lisoni J., Barlati S., Deste G., Ceraso A., Nibbio G., Baldacci G., Vita A. (2022). Efficacy and tolerability of Brain Stimulation interventions in Borderline Personality Disorder: State of the art and future perspectives—A systematic review. Prog. Neuro-Psychopharmacol. Biol. Psychiatry.

[99] Videler A.C., Hutsebaut J., Schulkens J.E.M., Sobczak S., van Alphen S.P.J. (2019). A Life Span Perspective on Borderline Personality Disorder. Curr. Psychiatry Rep..

